# A Systematic Review and Meta-Analysis of Efficacy of Botulinum Toxin A for Neuropathic Pain

**DOI:** 10.3390/toxins14010036

**Published:** 2022-01-03

**Authors:** Anupam Datta Gupta, Suzanne Edwards, Jessica Smith, John Snow, Renuka Visvanathan, Graeme Tucker, David Wilson

**Affiliations:** 1 Central Adelaide Rehabilitation Services, Queen Elizabeth Hospital, University of Adelaide, Adelaide, SA 5005, Australia; Jessica.Smith6@sa.gov.au; 2 Adelaide Health and Medical Sciences, University of Adelaide, Adelaide, SA 5005, Australia; Suzanne.Edwards@adelaide.edu.au; 3 Lyell McEwin Hospital, Northern Adelaide Local Health Network, Adelaide, SA 5112, Australia; John.Snow@sa.gov.au; 4 National Health and Medical Research Council Centre of Research Excellence in Frailty and Healthy Aging, Basil Hetzel Institute, 28 Woodville Road, Adelaide, SA 5011, Australia; Renuka.Visvanathan@adelaide.edu.au (R.V.); grtucker@adam.com.au (G.T.); David.Wilson@adelaide.edu.au (D.W.)

**Keywords:** neuropathic pain, botulinum toxin, systematic review, meta-analysis

## Abstract

We performed a systematic review and meta-analysis of randomised controlled trials (RCTs) conducted from January 2005 to June 2021 to update the evidence of Botulinum toxin A (BoNT-A) in neuropathic pain (NP) in addition to quality of life (QOL), mental health, and sleep outcomes. We conducted a Cochrane Grading of Recommendations Assessment, Development, and Evaluation (GRADE) criteria analysis of RCTs from the following data sources: EMBASE, CINAHL, WHO International Clinical Trial Registry Platform, ClinicalTrials.gov, Cochrane database, Cochrane Clinical Trial Register, Australia New Zealand Clinical Trials Registry, and EU Clinical Trials Register. Meta-analysis of 17 studies showed a mean final VAS reduction in pain in the intervention group of 2.59 units (95% confidence interval: 1.79, 3.38) greater than the mean for the placebo group. The overall mean difference for sleep, Hospital Anxiety and Depression Scale (HADS) anxiety, HADS depression, and QOL mental and physical sub-scales were, respectively, 1.10 (95% CI: −1.71, 3.90), 1.41 (95% CI: −0.61, 3.43), −0.16 (95% CI: −1.95, 1.63), 0.85 (95% CI: −1.85, 3.56), and −0.71 (95% CI: −3.39, 1.97), indicating no significance. BoNT-A is effective for NP; however, small-scale RCTs to date have been limited in evidence. The reasons for this are discussed, and methods for future RCTs are developed to establish BoNT-A as the first-line agent.

## 1. Introduction

Neuropathic pain (NP) resulting from damage or dysfunction of the peripheral or central nervous systems is one of the most common forms of pain, affecting up to 10% of the general population [1,2]. The current evidence of treatment outcomes puts success rates at best between 30% and 50%, and existing treatments come with significant side effects [3]. The effect of NP pervades patient sensation, thoughts, feelings, and behaviours. Treatment outcomes of these patients’ experiences have been described as “woefully inadequate” [1,3]. Clinicians’ express difficulty in dealing with NP [4], thus limiting the outcomes of pain relief, the healing of underlying conditions, and rehabilitation to a satisfactory functional quality of life.

**Figure 1:**
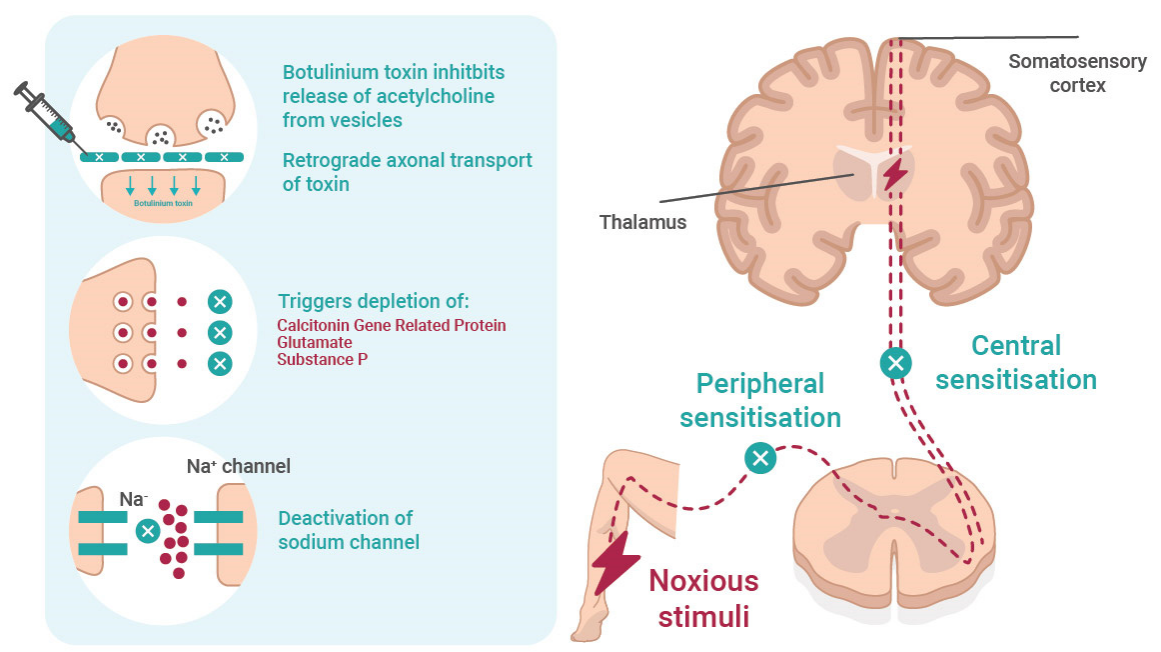
The mechanism and effect of botulinum toxin in neuropathic pain. Botulinum toxin reversibly inhibits the release of acetylcholine from the presynaptic vesicle and causes local chemodenervation resulting in reduced muscle contraction. The possible mechanisms of action on pain involves (i) retrograde axonal transport of toxin; (ii) inhibition of neuropeptides, such as substance P, calcitonin gene-related protein (CGRP), and glutamate; and (iii) deactivation of Na channel. All prevent peripheral and central sensitisation.

Over the last two decades, botulinum toxin A (BoNT-A) has found wider therapeutic acceptance in rehabilitation across a range of neurological disorders resulting in spasticity and/or dystonia and in other medical and surgical conditions [5,6]. Other scientific research from pharmacology, toxicology, and biology [6,7] has identified the mechanism of BoNT-A in NP (Figure 1) and supported its use as being safe and effective for an increasing number of applications, including a number of conditions with neuropathic pain. Multiple randomised controlled trials (RCTs) [8,9,10,11,12,13,14,15,16,17,18,19,20,21,22,23,24,25,26] and systematic reviews [27,28,29,30,31,32,33] have added evidence, specifically on NP. Despite this substantial body of evidence, BoNT-A has not been considered as adequate for the first-line treatment of NP, and it is reasonable and important for rehabilitation medicine physicians to ask why this is so.

To date, published RCTs on NP have shown efficacy and safety but have failed to provide adequate supportive evidence addressing the diagnosis of NP, the effective optimum dose, administration in different NP conditions, the duration of effectiveness, quality of life and functional ability, and other data that would underpin NP management guidelines. The limited design and small sample size of the RCTs led Shackleton et al. [28] to conclude that the promising pain control results shown in medical and surgical use in recent years required further well-designed placebo-controlled trials, not only to support BoNT-A as a backup treatment but also for first-line pain relief. Croford [3] has further argued that new treatments for chronic pain are of the utmost urgency. To design an appropriate guidelines trial, we require more detail on the limitations of the study evidence to date.

In Australia, a research evidence hierarchy has been developed by the National Health and Medical Research Council (NHMRC) [34]. This hierarchy assigns levels of evidence according to the type of research question and the quality of the research design appropriate to that question. RCTs are accepted as the building blocks of evidence required in the translation of research to wider medical practice. This can be followed by the Grading of Recommendations Assessment, Development, and Evaluation (GRADE) system [35], which is one of several systems for grading clinical evidence and creating clinical practice guidelines based on the underlying research evidence. To date, systematic reviews of NP have failed to achieve these research standards. It is the aim of this study to articulate this in detail by a more thorough analysis of the evidence provided to date.

A substantial review of 229 NP studies, updating pain research outcomes over the past ten years by Finnerup et al. [36], used the GRADE system to advise the Special Interest Group on NP regarding all treatments of NP. This review concluded that there was generally no evidence for the effectiveness of particular drugs in specific NP conditions and that an “inadequate response to NP drug therapy constitutes a highly unmet need that may have substantial consequences”. Its focus on BoNT-A was limited, but it did recommend BoNT-A as third-line treatment based on a small body of RCT evidence (six studies) [36]. 

Given the substantial promise of BoNT-A and the unmet need for better NP management, the present study aims to find a way forward that would improve the treatment and rehabilitation outcomes of NP. We first conduct an updated systematic review, adding five RCTs conducted since the previous review of Meng et al. in 2018 [27] to assess if anything has changed since that review. In this evidence upgrade, we make the immediate addition of RCTs considering both peripheral and central neuropathic pain, while previous reviews only considered peripheral neuropathic pain. 

Next, we conduct a qualitative analysis of included RCTs assessing the question and design limitations and the quality of the results, using established RCT design criteria. Given the severity of NP, its problems for both patients and clinicians, and the range of circumstantial evidence reported to date on the effectiveness, safety, and tolerability of BoNT-A, it is imperative to ask what is required in a well-designed RCT, as suggested by Shackleton et al. [28], which would underpin BoNT-A as the first-line treatment of NP.

## 2. Meta-Analysis Results

### 2.1. Final Visual Analogue Scale (VAS) Measures

The Visual Analog Scale (VAS) is discussed in some detail because of its consistent use as an outcome measure in the reviewed studies and, secondly, because of its importance of identifying bias in research and research reporting.

**Figure 2:**
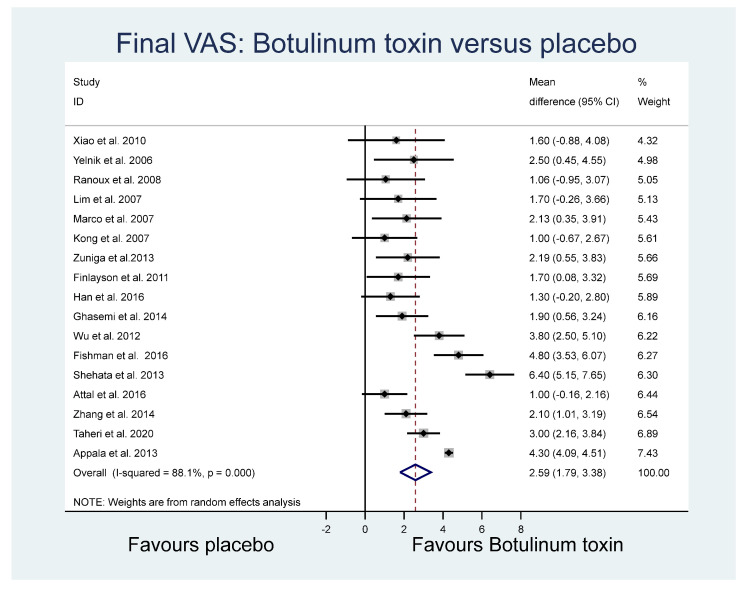
Final VAS.

The mean final VAS and the standard deviation of the final VAS for placebo and botulinum toxin groups were pooled across 17 studies using a random effects meta-analysis model. Heterogeneity in the study estimates was assessed using the I-squared statistic (88.1%) and Cochran’s Q *p* value (<0.0001), which showed considerable heterogeneity leading to the use of random effects models in analyses. The overall mean difference in the final VAS across the studies was 2.59 (95% confidence interval (CI): 1.79, 3.38) (Figure 2), identifying a mean higher VAS outcome pain score in the placebo group overall.

### 2.2. VAS Difference

The mean VAS differences (final minus baseline) and the standard deviation of VAS difference for the BoNT-A and placebo groups were pooled across 7 of the 17 studies. Heterogeneity analysis using the I^2^ statistic (88.2%) showed substantial heterogeneity. Overall, the mean difference in the final VAS units across the studies was 2.34 (95% CI: 1.07, 3.61), identifying a higher mean VAS outcome pain score in the placebo group.

### 2.3. 50% Reduction in VAS

The proportion of patients with at least a 50% reduction in VAS between baseline and final time periods in the BoNT-A and placebo groups was pooled across six studies using a random effects meta-analysis model. Heterogeneity in the study estimates was assessed using the I^2^ statistic (44.9%) and showed moderate heterogeneity. The mean relative risk across the studies was 4.90 (95% CI: 2.00, 6.13), identifying a higher mean VAS outcome pain score in the placebo group.

### 2.4. Pain Frequency (Number of Neuralgia Attacks in a Single Day)

The mean pain frequencies and standard deviations for the BoNT-A and placebo groups were pooled across three studies. Heterogeneity in the study estimates was assessed using the I^2^ statistic and showed moderate heterogeneity (49.7%). The overall mean difference in pain attacks across the studies was 24.47 (95% CI: 19.09, 29.86), identifying a higher mean frequency of pain in the placebo group.

### 2.5. Other Relevant Outcomes

**Figure 3:**
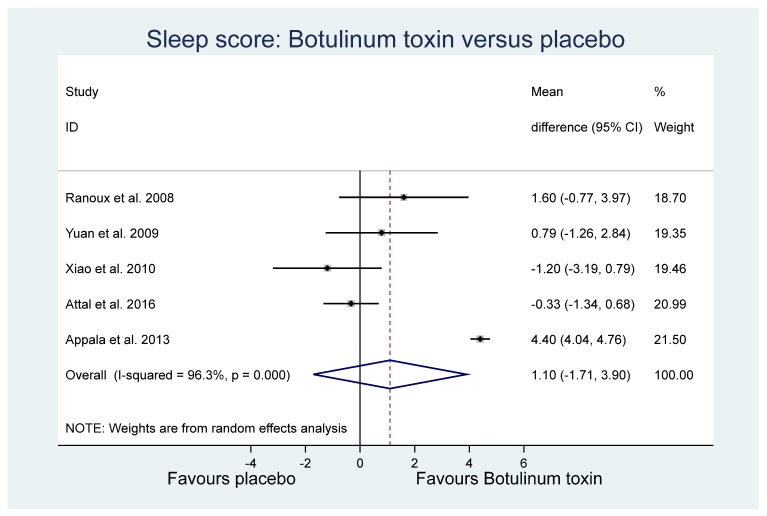
Sleep.

**Figure 4:**
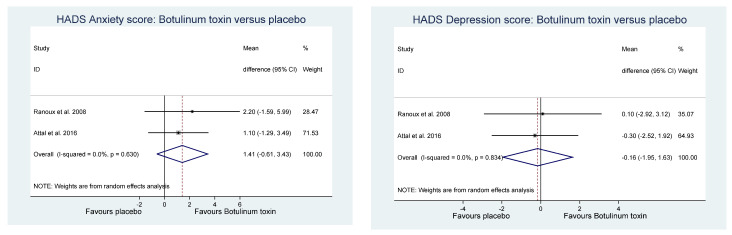
Hospital anxiety and depression score.

**Figure 5:**
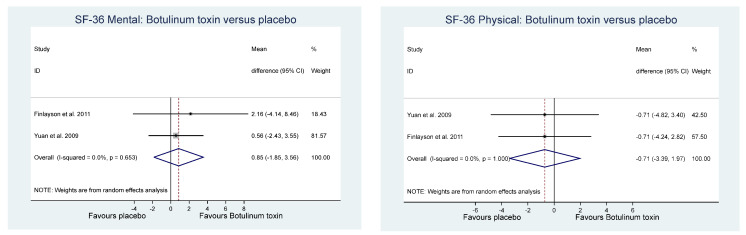
Quality of life.

Of the other study outcomes (sleep—three studies, anxiety—two studies, depression—two studies, and mental and physical health—two studies) included in the RCTs, there were no statistically significant differences observed between the BONT-A and placebo groups in the meta-analysis (Figure 3, Figure 4 and Figure 5).

### 2.6. Final VAS Funnel Plot

**Figure 6:**
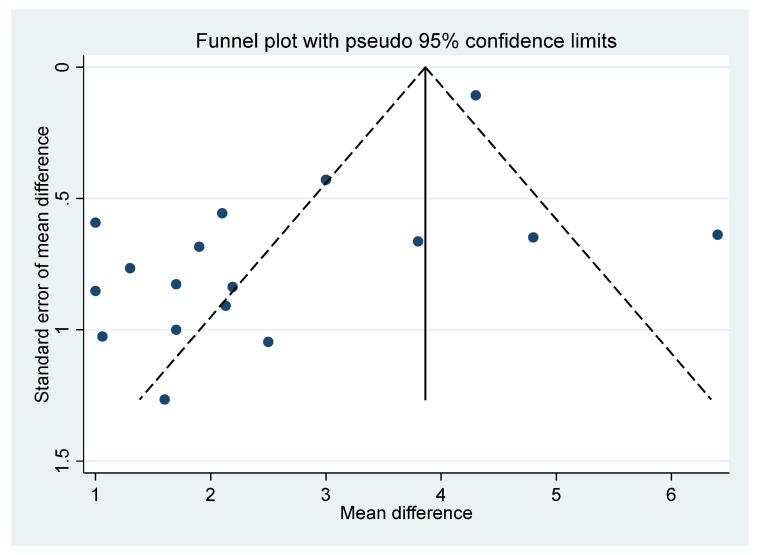
Funnel plot.

For the comparison of the BoNT-A and placebo final VAS (based on Figure 1), a funnel plot assessed publication bias for the final continuous VAS outcome (Figure 6). The funnel plot shows the standard error of mean difference on the *y*-axis and the mean difference on the *x*-axis. As most of the values fall outside the “funnel”, there is a substantial degree of study bias (Egger’s test: R = 2.73, *p* = 0.002). Oher likely sources of this bias are the small and variable sample sizes of the RCTs and the variability across other elements of RCT composition, including the sample (inpatients, outpatients, or both) [37]. 

## 3. Discussion

The main conclusion of this study is that although a substantial number of RCTs investigated BoNT-A’s efficacy in NP over time, the quality of the evidence produced does not support BoNT-A as the first-line treatment for NP. Apart from the review by Shackleton et al. [28], systematic reviews on the subject have not addressed the need to consider BoNT-A’s potential as first-line treatment. Given, however, that this study again finds that BoNT-A is effective in reducing pain and is seemingly durable without side effects, we conclude that an important armamentarium of neuropathic pain has languished [38,39]. For the rehabilitation medicine physicians engaged in neurorehabilitation, this is a major disadvantage given the current limiting success rates with standard treatments in pain management and the possibility that NP is likely to become a pathological condition for many patients. The complex peripheral and central mechanisms of neuropathic pain distinguish it from other types of pain in terms of its impact on functional ability, which is the primary outcome focus for the rehabilitation team dealing with NP. Although there is not enough evidence to elevate BoNT-A to first-line treatment status, there is enough evidence to identify that rehabilitation specialists may be missing out on an effective pharmacological intervention. This can only be assessed in an RCT meeting the approved research standards.

The issue of pain has been suggested as the fifth vital sign [40], yet, to date, the substantial systematic review evidence asserts that not much has changed in improving the management and outcomes of NP over the recent decades. The inter-disciplinary research evidence of BoNT-A’s analgesic effect and its antinociceptive and axonal transport properties [41] call for careful consideration of further research and the design of RCTs with NHMRC and GRADE quality. Such an RCT, besides the issues of effectiveness and safety, should include research questions that address specific guideline issues across neuropathic conditions, diagnosis, effective dosage, administration, and related pain outcome effects, in addition to appropriate design criteria and study samples as identified and reported in this paper. 

It is also important to reiterate that the RCTs conducted over the last two decades were largely repetitive in design and outcome, and they were based on small samples. We identify resource constraints as the probable reason. Despite this, we conclude that RCTs to date have failed to produce evidence that would appropriately assess the true value of BoNT-A for NP and the subsequent rehabilitation of individuals with NP. 

## 4. Conclusions

Two consistent conclusions can be drawn from this study. First, given the consistency of the VAS outcome measures used for most of the RCTs, BoNT-A had a statistically significant effect on pain consistent with previous systematic reviews. We can, again, reasonably conclude from the study reports that BoNT-A is safe for treating neuropathic pain, without notable side effects, and it is durable over an extended period. However, there is still a lack of satisfactory evidence from a high-quality RCT for translating evidence to practice. We believe further research is required in this area, which may lead to improved outcomes for the difficult problem that has languished for two decades. Based on this review, improved designs of future RCTs and resolution of the unanswered clinical questions are possible. The RCTs should also focus on the latency effect of BoNT-A in NP and perform a cost–benefit analysis for public health decisions. 

## 5. Methods

NP can be of peripheral or central origin, and, in upgrading the evidence base with recent publications, we added the latter category and included three publications on hemiplegic shoulder pain for this systematic review. Altogether, we produced meta-analyses for 17 RCTs, which is an addition of 5 RCTs to the most recent systematic review [27].

### 5.1. Search Methods

The literature on the effects of BoNT-A on NP was systematically reviewed. The data were collected for the previous fifteen years prior to June 2021.

### 5.2. Document Sources

Documents from Cochrane, EMBASE, CINAHL, and ProQuest (electronic databases online) were searched for relevant studies to identify RCTs conducted up until June 2021. 

### 5.3. Document Identification Methods

A detailed description of the search strategies is available on request. Key words used were “botulinum toxin”, “neuropathic pain”, and “neuralgia”, with filter settings for humans and the English language. The bibliographic references in the systematic reviews helped locate studies not found through database searches. Embase, ProQuest, EBM Reviews-Cochrane Central Register of controlled Trials, WHO International Clinical Trial Registry Platform (WHO-ICTRP), ClinicalTrials.gov, Cochrane Clinical Trial Register (CCTR), Australian New Zealand Clinical Trials Registry (ANZCTR), and EU Clinical Trials Register (EUCTR) were searched using the same combination of keywords for any unpublished RCTs missed during the search. 

### 5.4. Data Extraction

Data extracted from the study design and methods included the following: outcome, outcome measures, power, sample size, allocation concealment (blinding), entry criteria, pain diagnosis, pain source, dosage, study duration, statistical significance, randomisation, study duration, outcomes, outcome measures, and follow-up methods. 

### 5.5. Selection Methods of Included Documents in the Analysis

The selection of research materials was limited to full-text articles. The key words used had to be included in titles and abstracts, had to refer to randomised controlled trials using BoNT-A for NP, and had to be written in the last fifteen years in English. Articles selected were deemed appropriate by the study team if the studies included the RCT outcomes described above following BoNT-A injection for NP. 

### 5.6. Assessment of Methodological Quality 

The following study characteristics were considered consistent with Cochrane GRADE criteria: risk of bias, inconsistency, indirectness, imprecision, and publication bias. Two authors (Anupam Datta Gupta and Jessica Smith) independently assessed the quality of the evidence and resolved the disagreement with the third author (John Snow) to reach consensus.

### 5.7. Selecting Documents for Systematic Review

**Figure 7:**
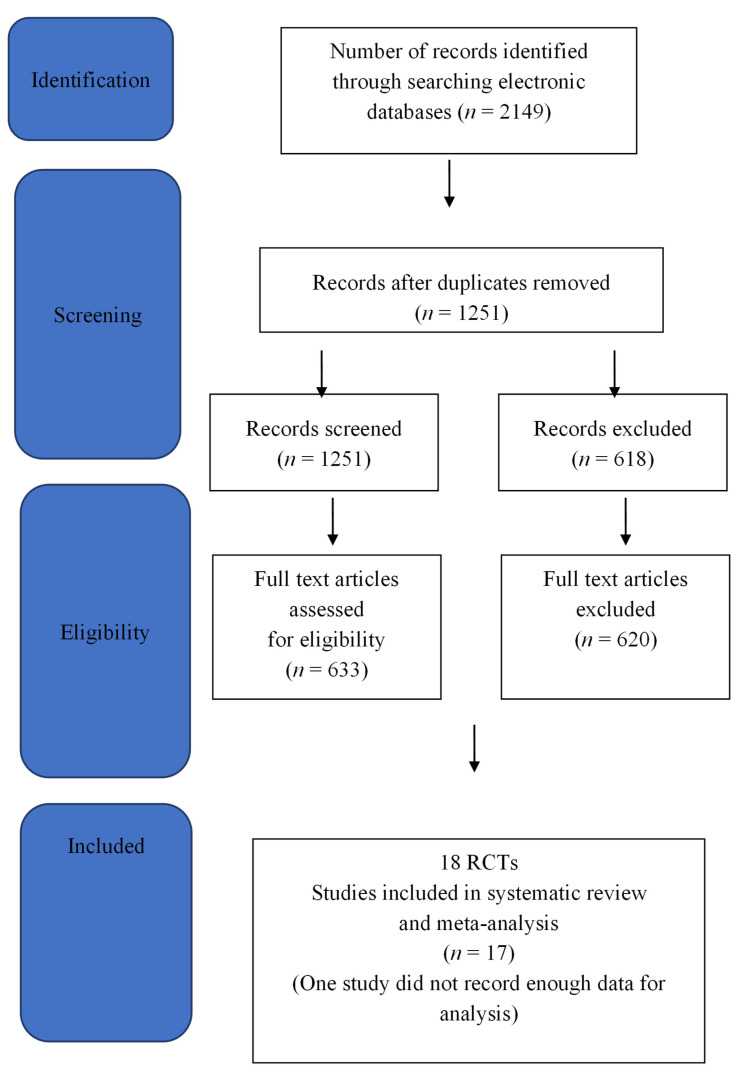
PRISMA diagram.

For the decision criteria (PRISMA) and results of the RCT studies appropriate for the systematic reviews and meta-analysis, see Figure 7.

A total of 2149 records were identified through database searches, and after duplicates were removed, 633 full text articles remained. Only 18 studies fulfilled selection criteria for a systematic review and meta-analysis. One RCT did not record the standard deviation or enough data to be included in the meta-analysis [25].

### 5.8. Qualitative Analysis

In a qualitative analysis of RCTs, we assessed how effectively the RCTs addressed the design assumptions and the quality of evidence in each study design. We concentrated on specific methodological issues of study design, addressing (i) the importance of the study questions and relationship to sample size; (ii) the sample quality and where the sample should be drawn; (iii) study error, bias, and confounding; (iv) fundamental limitations in study design; and (v) the critical outcome data. 

Further criteria used in assessing studies were informed by three scales previously used by Lee et al. to evaluate the quality of RCTs published in *The Clinical Journal of Pain* between 1997 and 2017 [37] (Jadad scale, van Tulder scale, and the Cochrane risk of bias tool), together with criteria from the American Academy of Neurology on classification of evidence schemes [37]. These criteria informed the study design items in the RCT quality analysis listed in Table 1. From Table 1, we ask where study quality can improve and what must be addressed in future BoNT-A studies if evidence is to contribute to guidelines and wider use for NP management in clinical medicine.

### 5.9. Statistical Methods

Data analyses were performed using Stata Statistical Software: Release 15.1 College Station, TX: StataCorp LP. In the meta-analysis, for continuous variables, mean differences with 95% confidence intervals (CIs) were calculated for each study and then for all the studies combined. For one binary outcome (50% reduction in VAS), relative risk and 95% CIs were calculated. A variable was included in the meta-analysis if at least 2 of the journal articles involved had sufficient values for that variable (e.g., mean and standard deviation in placebo and botulinum toxin groups). 

The I^2^ statistic [42] was used to evaluate the heterogeneity of RCTs (with I^2^ > 50% indicating significant study heterogeneity). In view of the heterogeneity found for several of the variables in this meta-analysis, a random effects model was used throughout. A funnel plot and Egger’s statistic [43] were also used to assess heterogeneity and the overall probability of publication/reporting and methodological bias of the RCT study results [44]. An asymmetrical funnel plot distribution indicates evidence of bias due to studies of less favourable effects not being published and/or poor methodological design [45]. 

### 5.10. RCT Quality Analysis

#### 5.10.1. Importance of the Study Questions and Relationship with Sample Size

Accurately defining the study question and outcome determines the sample size equation and the study power needed to detect an effect. Similar inter-relationships exist for other study criteria in Table 1. Incorrect design assumptions or processes at any stage of the design can affect subsequent design developments, thus compounding sources of bias in design logic. Multiple factors, including demographics, sample size, variance, power, dosage, and duration of studies, can influence the quality and magnitude of the meta-analysis, and the heterogeneity previously identified in the meta-analyses could relate to any of these factors.

In addition to the primary study questions of pain reduction, safety, and efficacy, only a small number of studies addressed other important indicators of pain reduction, including the outcomes of sleep and quality of life, and produced limited results that cannot be generalised. Study safety and efficacy were observed across the range of NP conditions studied. Beyond the fundamental question of pain reduction, studies were largely too underpowered to address other research questions and related design issues that would be required for the generalisability of BoNT-A’s use as the first-line treatment of NP. 

A small group of studies reported on the durability of BoNT-A, which, in one case, lasted up to six months. However, more specific outcome information is needed on the quality and range of durability based on BoNT-A dose to formulate guidelines. Dose titration is a critical concept affecting guidelines and information for practicing clinicians.

The issue of small sample size for each study identifies research work conducted with strictly limited resources, which constrains more complex study questions and study design in assessing pain outcomes. Despite this, most studies, again, concluded that the toxin was safe and effective, supporting the previous literature. 

Table 1 shows that RCT studies were diligent in addressing study entry criteria, confidence intervals of study effect, and the safety and tolerability of the toxin. 

#### 5.10.2. Sample Quality and Where the Sample Should Be Drawn

Calculation of sample size for most studies adopted a minimalist approach and was based solely on the ability to detect the difference in pain outcomes between BoNT-A and placebo. Nine studies provided inadequate information on study power and/or allocation concealment. Lee et al. points out that appropriate blinding and allocation concealment are associated with RCT quality [37]. 

In terms of sample source, all studies used inpatients and outpatients or both. Given that most people with chronic pain are seen in general practice and pain units, the use of inpatient/outpatient samples in RCTs are inappropriate, and future studies must draw from a more representative source (or both). Furthermore, inpatients and outpatients may represent a more severe pain group, and pain outcomes in this review may be unrepresentative overall. 

#### 5.10.3. Study Error, Bias, and Confounding

Several studies had no justification for sample size, and half of the RCTs did not adequately address blinding or allocation concealment, which, combined with other elements of bias, may add to the heterogeneity found in the meta-analyses. In a supplementary analysis using the interquartile range (IQR) of studies and adding 1.5 × IQR to the third quartile of the main meta data in Figure 2, we identified that four studies were outliers. If we were to remove these from that analysis, the risk ratio would be reduced from 2.59 to 1.68 (*p* > 0.05), thus considerably reducing confidence in efficacy in the VAS meta-analysis. It is also possible that, in some of the neuropathic conditions, there was the coexistence of both nociceptive and neuropathic pain. Given this possibility and the likelihood of other co-morbidities in older age groups, it is necessary to distinguish between pain categories at baseline and allow for these in study design analysis to prevent confounding. 

#### 5.10.4. Limitations in Study Design

It can also be seen from Table 1 that only six studies diagnosed NP; this needs to be the norm for future studies. All studies used the Visual Analogue Scale (or an equivalent measure) as the main outcome, raising the question of whether self-reported questionnaires of pain change are satisfactory evidence in assessing the treatment of NP. The self-reporting of pain can be affected by patients’ physiological and psychological status and underlying neuropathic condition, as well as the assessors’ predispositions [46]. For study precision, more substantial diagnostic measures of NP are available and should be used [47]. Bendiger and Plunkett [48] point out that NP is more distressing than other forms of pain and requires specialised diagnostic skills. As previously mentioned, a small number of studies addressed additional outcomes mainly covering sleep, mental health, and quality of life. All of these are appropriate additional outcomes expected from NP pain control, and the inclusion of these in future RCTs would notably contribute to supporting BoNT-A’s efficacy. 

The convenience sample approach used in the RCTs, and the heterogeneity found in this study, creates the possibilities of bias, confounding, and systematic error. An important aspect of sample size is that it cannot be reduced by simply increasing size. It must be reduced by a homogeneous sample of individuals with different NP conditions. A challenging question for future RCTs on BoNT-A in NP is just how broadly the sample and interventions should be defined. Such a study should be capable of addressing several study outcome measures, including optimum dose, duration of effectiveness, activities of daily living outcomes, and cost–benefit analysis. Studies should be adequately powered to support the study hypotheses regarding the use of BoNT-A for NP. Furthermore, these studies must change their focus to an adequate baseline diagnosis of NP, which is fundamental to the design of future RCTs.

## Figures and Tables

**Table 1:** Design characteristics of randomised controlled studies.

Authors/Year	Primary Outcome	Other Outcome	Sample Size	Power	Sample Source	Blinding	Entry Criteria	NP Diagnosed	Pain Source	BT Dose (Units)	SD/CI	Study Effect Sig.	Study Duration	*n* of Doses
Yelnik et al., 2006	VAS	No	*n* = 20	No	Not Given	DBND	Yes	No	HSP	500	Yes	Yes	4 weeks	1
Lim et al., 2008	NPS	No	*n* = 29	Yes	In/Out	DBND	Yes	No	HSP	100	Yes	Yes	12 weeks	1
Marco et al., 2007	VAS	No	*n* = 31	Yes	In	DBD	Yes	No	HSP	500	Yes	Yes	6 months	1
Kong et al., 2007	VAS	No	*n* = 17	Yes	Out	DBND	Yes	No	HSP	500	No	Yes	12 weeks	1
Ranoux et al., 2008	BPI VAS	No	*n* = 29	No	Pat	DBND	Yes	No	PHN, PT, PO	20–190	Yes	Yes	24 weeks	1
Yuan et al., 2009	VAS	Yes	*n* = 18	No	Com	DBND	Yes	Yes	DN	50	Yes	Yes	12 weeks	2
Xiao et al., 2010	VAS	Yes	*n* = 60	No	Ins	DBDD	Yes	No	PHN	200	No	Yes	3 months	1
Finlayson et al., 2011	VAS	No	*n* = 38	Yes	Out	DBD	Yes	Yes	PS	75	Yes	No	6 months	1
Wu et al., 2012	VAS	Yes	*n* = 42	No	Out	DBND	Yes	No	TN	100	No	Yes	13 weeks	1
Zuniga et al., 2013	VAS	No	*n* = 36	No	Out	DBD	nYes	No	PHN	50	Yes	Yes	12 weeks	1
Shehata et al., 2013	VAS	Yes	*n* = 20	No	Hos	DBD	Yes	Yes	TN	100	Yes	Yes	12 weeks	1
Apalla et al., 2013	VAS	No	*n* = 30	Yes	Out	DBD	Yes	No	PHN	100	Yes	Yes	16 weeks	1
Ghasemi et al., 2014	NPS	Yes	*n* = 40	No	Com	DBND	Yes	Yes	DN	100	Yes	Yes	3 weeks	1
Zhang et al., 2014	VAS	No	*n* = 84	No	Out/In	DBD	Yes	No	TN	25 & 75	Yes	Yes	9 weeks	1
Han et al., 2016	VAS	No	*n* = 40	Yes	Com	DBD	Yes	No	SCI	200	Yes	Yes	8 weeks	1
Fishman et al., 2017	VAS	No	*n* = 28	No	Out	DBND	Yes	Yes	PS	300	Yes	Yes	12 weeks	1
Taheri et al., 2020	VAS	No	*n* = 141	Yes	Out	DBD	Yes	Yes	DN	150	Yes	Yes	16 weeks	1

NPS—numeric pain scale, BPI—Brief Pain Inventory, DBD—double blind discussed, DBND—double blind not discussed, In—inpatients, Out—outpatients, NP—neuropathic pain, PHN—post-herpetic neuralgia, HSP—hemiplegic shoulder pain, PT—post-traumatic, PO—post-operative, PS—pyriformis syndrome, SCI—spinal cord injury, DN—diabetic neuropathy, TN—trigeminal neuralgia. VAS—Visual Analogue Scale, SD—standard deviation, CI—confidence interval.

## Data Availability

Data available on request.

## References

[1] Smith B.H., Hébert H.L., Veluchamy A. (2020). Neuropathic pain in the community: Prevalence, impact, and risk factors. Pain.

[2] Wan A. (2014). GP pain management: What are the P’s and A’s of pain management. Aust. Fam. Physician.

[3] Crofford L.J. (2015). Chronic Pain: Where the Body Meets the Brain. Trans. Am. Clin. Climatol. Assoc..

[4] Upshur C.C., Luckmann R.S., Savageau J.A. (2006). Primary care provider concerns about management of chronic pain in community clinic populations. J. Gen. Intern. Med..

[5] Intiso D. (2012). Therapeutic Use of Botulinum Toxin in Neurorehabilitation. J. Toxicol..

[6] Gooriah R., Ahmed F. (2015). Therapeutic uses of botulinum toxin. J. Clin. Toxicol..

[7] Pirazzini M., Rossetto O., Eleorpra R., Montecucco Witkin J.M. (2017). Botulinum neurotoxins: Biology, pharammcologyand toxicology. Pharmacol. Rev..

[8] Yuan R.Y., Sheu J.J., Yu J.M., Chen W.T., Tseng I.J., Chang H.H., Hu C.J. (2009). Botulinum toxin for diabetic neuropathic pain: A randomized double-blind crossover trial. Neurology.

[9] Yelnik A.P., Colle F.M., Bonan I.V., Vicaut E. (2007). Treatment of shoulder pain in spastic hemiplegia by reducing spasticity of the subscapular muscle: A randomised, double blind, placebo controlled study of botulinum toxin A. J. Neurol. Neurosurg. Psychiatry.

[10] Marco E., Duarte E., Vila J., Tejero M., Guillen A. (2007). Is Botulinum Toxin Type A Effective in The Treatment of Spastic Shoulder Pain in Patients After Stroke Unclear Risk. A Double-blind Randomized Clinical Trial. J. Rehabil. Med..

[11] Kong K.-H., Neo J.-J., Chua K.S. (2007). A randomized controlled study of botulinum toxin A in the treatment of hemiplegic shoulder pain associated with spasticity. Clin. Rehabil..

[12] Lim J.-Y., Koh J.-H., Paik N.-J. (2008). Intramuscular Botulinum Toxin-A Reduces Hemiplegic Shoulder Pain. Stroke.

[13] Ranoux D., Attal N., Morain F., Bouhassira D. (2008). Botulinum toxin type a induces direct analgesic effects in chronic neuropathic pain. Ann. Neurol..

[14] Xiao L., Mackey S., Hui H., Xong D., Zhang Q., Zhang D. (2010). Subcutaneous Injection of Botulinum Toxin A Is Beneficial in Postherpetic Neuralgia. Pain Med..

[15] Finlayson H.C., O’Connor R.J., Brasher P.M., Travlos A. (2011). Botulinum toxin injection for management of thoracic thoracic outlet syndrome: A double-blind, randomized trial. Pain.

[16] Wu C.-J., Lian Y.-J., Zheng Y.-K., Zhang H.-F., Chen Y., Xie N.-C., Wang L.-J. (2012). Botulinum toxin type A for the treatment of trigeminal neuralgia: Results from a randomized, double-blind, placebo-controlled trial. Cephalalgia.

[17] Shehata H.S., El-Tamawy M.S., Shalaby N.M., Ramzy G. (2013). Botulinum toxin-type A: Could it be an effective treatment option in intractable trigeminal neuralgia?. J. Headache Pain.

[18] Apalla Z., Sotiriou E., Lallas A., Lazaridou E., Loannides D. (2013). Botulinum Toxin A in Postherpetic Neuralgia A Parallel, Randomized, Double-Blind, Single-Dose, Placebo-controlled Trial. Clin. J. Pain.

[19] Zúñiga C., Piedimonte F., Díaz S., Micheli F. (2013). Acute Treatment of Trigeminal Neuralgia With Onabotulinum Toxin A. Clin. Neuropharmacol..

[20] Ghasemi M., Ansari M., Basiri K., Shaygannejad V. (2014). The effects of intradermal botulinum toxin type a injections on pain symptoms of patients with diabetic neuropathy. J. Res. Med. Sci..

[21] Zhang H., Lian Y., Ma Y., Chen Y., He C., Xie N., Wu C. (2014). Two doses of botulinum toxin type A for the treatment of trigeminal neuralgia: Observation of therapeutic effect from a randomized, double-blind, placebo-controlled trial. J. Headache Pain.

[22] Han Z., Song D.H., Oh H., Chung M.E. (2016). Botulinum toxin type A for neuropathic pain in patients with spinal cord injury. Ann. Neurol..

[23] Attal N., de Andrade D.C., Adam F., Ranoux D., Teixeira M.J., Galhardoni R., Raicher I., Üçeyler N., Sommer C., Bouhassira D. (2016). Safety and efficacy of repeated injections of botulinum toxin A in peripheral neuropathic pain (BOTNEP): A randomised, double-blind, placebo-controlled trial. Lancet Neurol..

[24] Fishman L.M., Wilkins A.N., Rosner B. (2017). Electro physiologically identified piriformis syndrome is successfully treated with incobotulinum toxin and physical therapy. Muscle Nerve.

[25] Salehi H., Moussaei M., Kamiab Z., Vakilian A. (2019). The effects of botulinum toxin type A injection on pain symptoms, quality of life, and sleep quality of patients with diabetic neuropathy: A randomized double-blind clinical trial. Iran. J. Neurol..

[26] Taheri M., Sedaghat M., Solhpour A., Rostami P., Lima B.S. (2020). The Effect of Intradermal Botulinum Toxin a injections on painful diabetic polyneuropathy. Diabetes Metab. Syndr..

[27] Meng F., Peng K., Yang J.-P., Ji F.-H., Xia F., Meng X.-W. (2018). Botulinum toxin-A for the treatment of neuralgia: A systematic review and meta-analysis. J. Pain Res..

[28] Shackleton T., Ram S., Black M., Ryder J., Clark G.T., Enciso R. (2016). The efficacy of botulinum toxin for the treatment of trigeminal ad postherpetic neuralgia: A systematic review with meta-analyses. Oral Surgery Oral Med. Oral Path. Oral Rad..

[29] Morra M.E., Elgebaly A., Elmaraezy A., Khalil A.M., Altibi A.M.A., Vu T.L.-H., Mostafa M.R., Huy N.T., Hirayama K. (2016). Therapeutic efficacy and safety of botulinum toxin A therapy in trigeminal neuralgia: A systematic review and meta-analysis of randomised controlled trials. J. Headache Pain.

[30] Nauman M., Jankovic J. (2004). Safety of botulinum toxin type A: A systematic review and meta-analysis. Curr. Med. Res. Opin..

[31] Herd C.P., Tomlinson C.L., Rick C., Scotton W.J., Edwards J., Ives N.J., Clarke C., Sinclair A.J. (2019). Cochrane systematic review and meta-analysis of botulinum toxin for the prevention of migraine. BMJ Open.

[32] Lakhan S.E., Velasco D.N., Tepper D. (2015). Botulinum Toxin-A for Painful Diabetic Neuropathy: A Meta-Analysis. Pain Med..

[33] Wei J., Zhu X., Yang G., Shen J., Xie P., Zuo X., Xia L., Han Q., Zhao Y. (2019). The efficacy and safety of botulinum toxin type A treatment of trigeminal neuralgia and peripheral neuropathic pain: A meta-analysis of randomised controlled trails. Brain Behav..

[34] NHMRC Levels of Evidence and Grades for Recommendations for Developers of Guidelines. https://www.nhmrc.gov.au/sites/default/files/images/NHMRC%20Levels%20and%20Grades%20(2009).pdf.

[35] Guyatt G., Oxman A.D., Akl E.A., Kunz R., Vist G., Brozek J., Norris S., Falck-Ytter Y., Glasziou P., DeBeer H. (2011). GRADE guidelines: 1. Introduction—GRADE evidence profiles and summary of findings tables. J. Clin. Epidemiol..

[36] Finnerup N.B., Attal N., Haroutounian S., McNicol E., Baron R., Dworkin R.H., Gilron I., Haanpää M., Hansson P., Jensen T.S. (2015). Pharmacotherapy for neuropathic pain in adults: A systematic review and meta-analysis. Lancet Neurol..

[37] Lee S.W., Kim K.S., Chung J.H., Park H.J., Sohn Y.S., Jeon W.J., Cho S.Y., Shin W.J. (2012). Assessing the quality of randomised controlled trails of complex regional pain syndrome published in the Journal of Clinical Pain Medicine Field. J. Korean Med. Sci..

[38] Egeo G., Fofi L., Barbanti P. (2020). Botulinum Neurotoxin for the Treatment of Neuropathic Pain. Front. Neurol..

[39] Datta Gupta A., Wilson D.H. (2018). Spasticity/Dystonia—Current Pharmaceutical Benefits Scheme-Guidelines for Treatment with Botulinum Toxin—A Case for Change. Med. J. Aust..

[40] Levy N., Sturgess J., Mills P. (2018). “Pain as the fifth vital sign” and dependence on the “numerical pain scale” is being abandoned in the US: Why?. Br. J. Anaesth..

[41] Matak I., Riederer P., Lackovic Z. (2012). Botulinum toxin’s axonal transport properties from periphery to spinal cord. Neurochem. Int..

[42] Sterne J.A.C., Sutton A.J., Ioannidis J.P.A., Terrin N., Jones D.R., Lau J., Carpenter J., Rucker G., Harbord R.M., Schmid C.H. (2011). Recommendations for examining and interpreting funnel plot asymmetry in meta-analyses of randomised controlled trials. BMJ.

[43] Egger M., Smith G.D., Schneider M., Minder C. (1997). Bias in meta-analysis detected by a simple, graphical test. BMJ.

[44] Cochrane Handbook for Systematic Reviews of Interventions, Version 5.1.0 edited by Julian PT and Sally Green. https://handbook-5-1.cochrane.org/.

[45] Song F., Khan K.S., Dinnes J., Sutton A.J. (2002). Asymmetric funnel plots and publication bias in meta-analyses of diagnostic accuracy. Int. J. Epidemiol..

[46] Lucas R.E., Diener E., Oishi S., Tay L. (2018). Re-evaluating the strengths and weaknesses of self-report measures of subjective well-being. Handbook of Well-Being.

[47] Smith B.H., Torrance N., Ferguson J.A., Bennett M.I., Serpell M.G., Dunn K.M. (2012). Towards a definition of refractory neuropathic pain for epidemiological research. An international Delphi survey of experts. BMC Neurol..

[48] Bendiger T., Plunkett N. (2016). Measurement of pain medicine. An international Delphi Survey of Experts. BJA Educ..

